# Associations between metabolic traits and gout risk

**DOI:** 10.1097/MD.0000000000044101

**Published:** 2025-08-22

**Authors:** Jiayi Sun, Yingchao Guan, Dong Zhao, Sixuan Du, Wenhui Hao

**Affiliations:** a Department of Biochemistry and Molecular Biology, School of Basic Medical Sciences, Xinjiang Medical University, Urumqi, Xinjiang, China; b Changji People’s Hospital, Changji, Xinjiang, China.

**Keywords:** gout, lipids, Mendelian randomization, metabolic traits, UK Biobank

## Abstract

Gout is an inflammatory condition primarily driven by elevated serum urate levels, with metabolic factors playing key roles. Mendelian randomization (MR) was employed to assess the causal impact of 249 specific metabolic traits on gout risk. We used data from the UK Biobank as exposures and conducted a MR analysis with gout as the outcome of a large-scale association study. The inverse-variance-weighted method served as the primary analytical approach, supplemented by the weighted median and MR-Egger techniques. False discovery rate corrections and the MR Pleiotropy Residual Sum and Outlier method were applied to ensure robust results. Significant associations were found for alanine levels, glycoprotein acetyls, ratio of cholesteryl esters to total lipids in medium very low-density lipoprotein, ratio of triglycerides to phosphoglycerides, and triglyceride to total lipid ratio in very large high-density lipoprotein, indicating that these metabolic traits are causally linked to gout. Our findings confirmed the causal relationships between certain metabolic profiles and the risk of developing gout, highlighting potential targets for preventive and therapeutic interventions.

## 1. Introduction

Gout, a common form of inflammatory arthritis, manifests as acute and often excruciating pain, usually in the joints of the extremities and most characteristically in the big toe.^[1]^ This condition is primarily driven by hyperuricemia, the elevated levels of uric acid in the blood, which crystallize and deposit in joints, tendons, and surrounding tissues.^[2,3]^ Despite a well-established link between urate metabolism and gout, emerging evidence suggested that broader metabolic dysfunction may significantly contribute to the pathogenesis of the disease.^[4]^

Recent epidemiological studies highlighted the potential role of various metabolic factors, including lipids and amino acids, in influencing the risk of gout.^[5]^ For instance, elevated levels of certain lipoproteins were associated with increased gout incidence, suggesting a complex interplay between lipid metabolism and urate crystal deposition.^[6]^ Additionally, deviations in amino acid profiles were linked to altered renal and hepatic functions, which were crucial for urate excretion and gout pathology.

To address the gaps in understanding the causal relationships between these metabolic traits and gout, we conducted a Mendelian randomization (MR) study using 249 metabolites from the UK Biobank as exposures and gout derived from an association analysis as the outcome. The selection of the 249 metabolic traits in our study was guided by their established biological relevance to uric acid metabolism, inflammation, and lipid homeostasis, which are critical aspects of gout pathophysiology. These metabolites were chosen based on prior evidence from genome-wide association studies (GWAS) linking them to hyperuricemia and gout. For instance, certain amino acids like alanine may influence uric acid synthesis or renal excretion, while specific lipid ratios could reflect disturbances in lipid transport or storage that predispose individuals to gout. Additionally, inflammatory markers such as glycoprotein acetyls were associated with the inflammatory processes in gout. The UK Biobank’s comprehensive metabolic profiling protocol also played a role in guiding the selection of these traits. MR inferred causal relationships between exposures and outcomes by utilizing genetic variations that are randomly assigned at conception and persist throughout an individual’s life as instrumental variables (IVs), thereby effectively simulating a randomized controlled trial.^[7]^ Recent methodological advancements in MR analysis for complex diseases further reinforce its utility in disentangling causal pathways.^[8]^

## 2. Materials and methods

The following theories form the basis of MR analysis: there is no direct association between IVs and outcomes; outcomes are only indirectly influenced by their impact on exposure. IVs were correlated with exposure; no relationship existed between the IVs and any potential confounding variables.

### 2.1. Source of data

In this investigation, 249 metabolites, such as lipoproteins, cholesterol, unsaturated fatty acids, phospholipids, and amino acids, were employed as exposures and were sourced from the UK Biobank by Richardson et al Gout, which served as the outcome, originated in the FinnGen Biobank.^[9]^ The summary-level GWAS data for these exposures and outcomes were available on the Open GWAS project and FinnGen Biobank. Table 1 provides further details regarding exposure and outcomes. This investigation used data from public databases, which were anonymized and publicly available, and hence, did not require an ethics review.

**Table 1:** Detailed information of gout outcome and metabolic exposures.

Exposure/ outcome	Exposure or outcome names	Phenocode in IEU OpenGWAS project or the Finngen Biobank.	Sample size	The article or biobank of the data source	Number of cases	Number of controls
Exposure	249 metabolic traits	ebi-a-GCST90092803–ebi-a-GCST90093051	110,051–115,082	The UK Biobank (PMID: 35213538)	NA	NA
Outcome	Gout	M13_GOUT	40,266	The Finngen Biobank	9568	262,844

More information about exposures and outcome is available at the IEU OpenGWAS project (https://gwas.mrcieu.ac.uk/) and (https://r10.risteys.finngen.fi/).

GWAS = genome-wide association studies, IEU = Integrative Epidemiology Unit, NA = not applicable.

### 2.2. IVs selection

MR studies employed single-nucleotide polymorphisms (SNPs) as IVs to infer causal relationships between exposures and outcomes. The criteria used to select SNPs included a clumping window larger than 10,000 kb, linkage disequilibrium level <0.001 (*r*^2^ < 0.001), and significance level below the genome-wide threshold (*P* < 5 × 10^-8^). To minimize potential bias and avoid violating the assumptions of MR, SNPs that were significantly associated with the outcome (*P* < 5 × 10^-8^) were excluded from each analysis.

### 2.3. Statistical analysis

This study employed 3 commonly used MR analysis approaches to evaluate the causal linkages. Among these, the inverse-variance-weighted (IVW) approach is the primary method used to infer causal linkages in this investigation, as it is more robust than both the weighted median and MR-Egger approaches.^[10]^ For the IVW technique to be effective, horizontal pleiotropy must be either negligible or balanced. We utilized the MR-Egger technique to evaluate horizontal pleiotropy, taking advantage of its capacity to accommodate nonzero intercepts. We employed false discovery rate (FDR) corrections to reduce the FDR associated with the *P*-values generated by the IVW technique in multiple hypothesis-testing procedures. We applied the MR Pleiotropy Residual Sum and Outlier technique to detect the outliers. Upon detecting any outliers, we reran all analyses after excluding them. Cochran test was used to assess heterogeneity. When heterogeneity was detected (*P* < .05), the random-effects IVW model was applied; otherwise (*P* ≥ .05), the fixed-effects IVW model was applied. All statistical analyses were conducted using R (version 4.3.2, R Foundation for Statistical Computing, Vienna, Austria) and the TwoSampleMR package (version 0.5.10, MRC Integrative Epidemiology Unit, University of Bristol, Bristol, United Kingdom).^[11]^ SNPs with missing allele frequency > 5% were excluded. Outliers in metabolite levels (±4 standard deviation [SD] from mean) were trimmed. Horizontal pleiotropy was assessed via MR-Egger intercept tests (FDR < 0.05). MR Pleiotropy Residual Sum and Outlier identified and removed 3 outliers.

## 3. Results

This MR study assessed the causal effects of 249 metabolic traits on gout. Table 1 shows detailed results concerning the association between 249 metabolic traits and gout across 9568 cases, 262,844 controls, and over 100,000 participants per trait. Additional data could be accessed in the UK Biobank or in Table S1, Supplemental Digital Content, https://links.lww.com/MD/P774 of the referenced study. The primary analysis utilized the IVW method, supported by MR-Egger and weighted median methods to account for potential horizontal pleiotropy.

### 3.1. Metabolic traits and gout susceptibility

Significant metabolic predictors of gout were identified and the results are presented in Table 2. Key findings include the following: alanine levels showed a positive association with gout risk (IVW β = 0.204, SE = 0.100, *P* = .041), suggesting that higher alanine concentrations may increase the likelihood of developing gout. Glycoprotein acetyl levels were also positively associated with gout (IVW β = 0.173, SE = 0.078, *P* = .027), highlighting a novel biomarker for potential intervention. The lipid ratios provided significant insights, with the ratio of triglycerides to phosphoglycerides showing a particularly strong association (IVW β = 0.172, SE = 0.072, *P* = .017). The β-coefficient for alanine (0.204) corresponds to a 22.7% increased gout risk per SD increase (OR = 1.23, 95% CI:1.01–1.49), comparable to obesity-associated risk (OR = 1.3–1.5). For triglyceride to phosphoglyceride ratio (β = 0.172), each SD increase equates to 18.8% higher risk.

**Table 2:** Key metabolic indicators linked to gout risk.

Outcome	Exposure	Number of SNPs	IVW β	IVW SE	IVW *P*-value
Gout ‖ id: M13_GOUT	Alanine levels ‖ id: ebi-a-GCST90092806	28	0.204	0.100	.041
Gout ‖ id: M13_GOUT	Glycoprotein acetyls levels ‖ id: ebi-a-GCST90092821	47	0.173	0.078	.027
Gout ‖ id: M13_GOUT	Cholesteryl esters to total lipids ratio in medium VLDL ‖ id: ebi-a-GCST90092919	58	-0.127	0.064	.046
Gout ‖ id: M13_GOUT	Ratio of triglycerides to phosphoglycerides ‖ id: ebi-a-GCST90092983	62	0.172	0.072	.017
Gout ‖ id: M13_GOUT	Triglycerides to total lipids ratio in very large HDL ‖ id: ebi-a-GCST90093015	54	0.157	0.074	.035

β-values represent change in log-odds of gout per SD increase in metabolite level.

FDR = false discovery rate-adjusted *P*-value, HDL = high-density lipoprotein, SD = standard deviation, SE = standard error, SNPs = single-nucleotide polymorphisms, VLDL = very low-density lipoprotein.

### 3.2. Specific lipid associations

The cholesteryl ester-to-total lipid ratio in medium very low-density lipoprotein (VLDL) demonstrated an inverse association with gout (IVW β = −0.127, SE = 0.064, *P* = .046), suggesting that alterations in lipid processing or storage may influence gout pathogenesis. Similarly, the triglyceride to total lipid ratio in very large high-density lipoprotein was significantly associated with gout risk (IVW β = 0.157, SE = 0.074, *P* = .035), indicating a complex relationship between high-density lipoprotein subfractions and gout. The details are presented in Table 1.

### 3.3. Pleiotropy and sensitivity analysis

No evidence of widespread pleiotropy was detected via MR-Egger intercepts, confirming the specificity of metabolic traits in relation to gout. Sensitivity analyses using the weighted median method generally corroborated the IVW findings and underscored the robustness of the results.

### 3.4. Visualization of data

Figure S1, Supplemental Digital Content, https://links.lww.com/MD/P775, graphically represents the effect sizes and confidence intervals for all metabolic traits, facilitated visual appreciation of the breadth and implications of the data.

## 4. Discussion

This MR study elucidated the complex metabolic underpinnings of gout, revealing significant associations with specific metabolic traits, notably amino acids, and various lipid ratios. These findings, derived from a robust analysis involving 9568 gout cases and 262,844 controls from the UK and FinnGen Biobanks, provide new insights into potential metabolic targets for gout prevention and treatment.

Our findings revealed that elevated levels of alanine were associated with an increased risk of gout. This association provides compelling evidence that amino acid metabolism plays a significant role in the pathogenesis of this condition. Alanine, a nonessential amino acid, may influence uric acid synthesis and renal excretion through its role in energy metabolism and insulin resistance. Specifically, insulin resistance, which is linked to alanine metabolism, can lead to decreased ATP levels within cells, thereby increasing the production of xanthine and hypoxanthine, precursors of uric acid.^[12,13]^ This association suggests that interventions targeting amino acid metabolism may provide new avenues for managing hyperuricemia and gout. Further biochemical studies are required to elucidate the mechanisms by which alanine influences uric acid levels, potentially offering a novel biomarker for early detection and management strategies in at-risk populations. The association of glycoprotein acetylases with gout suggests a role for protein glycosylation in inflammatory processes related to gout. Glycosylation affects protein function and cellular interactions, which were critical for immune response and inflammation.^[14,15]^ Metabolomic insights into glycoprotein acetylation align with broader evidence linking metabolic dysregulation to inflammatory diseases.^[16]^ Since gout is characterized by an intense inflammatory response to urate crystals, understanding how glycosylation patterns might influence this process could reveal new therapeutic targets. Modulation of glycoprotein acetylation could potentially alter the inflammatory response in gout, offering a novel approach to treatment that needs to be explored in future clinical trials. The significant findings regarding lipid ratios, particularly the triglyceride to phosphoglyceride ratio and the cholesteryl ester to total lipid ratio in medium VLDL, highlight the complex interplay between lipid metabolism and gout. These lipid ratios may reflect underlying disturbances in lipid transport mechanisms or lipoprotein behavior that predispose individuals to gout.^[17]^ For instance, the triglyceride to phosphoglyceride ratio could indicate an altered lipid profile that enhances the solubility of urate in the blood, thereby increasing the risk of crystal formation. Furthermore, the inverse association of cholesteryl esters in medium VLDL with gout risk suggests that specific lipoprotein particles may exert protective effects against gout. This could be related to their role in the inflammatory pathways, as VLDL particles have been implicated in the modulation of inflammation.^[18,19]^ These findings suggest that lipid-lowering therapies may also benefit gout management. However, the specific mechanisms by which these lipid fractions influence gout risk remain to be clarified, necessitating detailed mechanistic studies. Although these metabolites were not yet used as clinical biomarkers, our findings provide new insights into potential metabolic targets for gout prevention and treatment. These insights into lipid metabolism emphasize the need for a broader approach to gout management, which includes monitoring and modifying lipid profiles. Lifestyle interventions such as dietary modifications and physical activity, which were known to influence lipid levels, could be particularly effective in managing gout risk.^[20,21]^ This approach also calls for a personalized medical strategy, in which lipid-modifying treatments might be tailored based on individual risk profiles identified through such metabolic markers. In conclusion, expanding our understanding of the metabolic contributors to gout, such as amino acid and lipid ratios, not only enhances our understanding of its pathophysiology but also opens up new potential therapeutic avenues. Future research should focus on integrating these metabolic insights into clinical practice, potentially revolutionizing gout management.

The strengths of this study are its large sample size and the application of rigorous MR techniques, which minimize confounding and reverse causation. However, the findings should be interpreted considering potential limitations, such as residual confounding by unmeasured factors and the assumption of linear effects in MR analysis. Although MR is a powerful tool to infer causality, it relies on several key assumptions, including the relevance, validity, and independence of the instrumental variables. Any violation of these assumptions could potentially bias the results. For example, if the selected SNPs (instrumental variables) are associated with other confounding factors or have pleiotropic effects on the outcome through pathways unrelated to the exposure, it could lead to biased estimates. While we employed methods like MR-Egger and the weighted median to address potential pleiotropy, we cannot entirely rule out the possibility of residual pleiotropy. Additionally, the selection of instrumental variables is crucial, and if the chosen SNPs do not adequately capture the variation in the exposure or are not strongly associated with it, it could reduce the power of the analysis and affect the reliability of the causal estimates. Furthermore, the possibility of horizontal pleiotropy, although addressed through sensitivity analysis, cannot be entirely excluded. These observed associations warrant further investigation through biochemical studies to elucidate the precise mechanisms by which these metabolic alterations contribute to gout.^[21,22]^ While our MR analysis provided causal evidence, the cross-sectional nature of the exposure–outcome data limits insights into the long-term dynamics of metabolic traits and gout progression. Our findings complement recent MR studies on gout pathogenesis, which similarly identified lipid metabolism as a key modulator of urate dynamics.^[23]^ Longitudinal cohort studies tracking metabolic profiles and incident gout cases over time are essential to validate these findings and clarify whether the identified associations reflect persistent risk factors or transient metabolic states. Additionally, expanding research on diverse populations will also be crucial for validating and generalizing these findings, ensuring that therapeutic strategies are effective across different genetic backgrounds.

## 5. Conclusions

This study provided compelling evidence linking specific metabolic traits to the risk of gout and highlights potential targets for therapeutic intervention. These insights into the metabolic pathways involved in gout opened new avenues for research, and offered hope for developing more effective prevention and treatment strategies tailored to individual metabolic profiles.

## Author contributions

**Conceptualization:** Jiayi Sun, Wenhui Hao.

**Data curation:** Jiayi Sun, Wenhui Hao.

**Formal analysis:** Jiayi Sun, Yingchao Guan, Sixuan Du, Wenhui Hao.

**Funding acquisition:** Wenhui Hao.

**Investigation:** Wenhui Hao.

**Methodology:** Jiayi Sun, Yingchao Guan, Dong Zhao, Sixuan Du.

**Project administration:** Dong Zhao, Wenhui Hao.

**Resources:** Wenhui Hao.

**Software:** Jiayi Sun.

**Supervision:** Wenhui Hao.

**Validation:** Dong Zhao, Wenhui Hao.

**Visualization:** Jiayi Sun, Yingchao Guan, Sixuan Du, Wenhui Hao.

**Writing – original draft:** Jiayi Sun.

**Writing – review & editing:** Jiayi Sun, Yingchao Guan, Sixuan Du, Wenhui Hao.

## Supplementary Material


