# Supplementary material for: Associations between metabolic traits and gout risk
Source: Medicine (Baltimore). 2025 Aug 22;104(34):e44101. doi: 10.1097/MD.0000000000044101 (PMC12384979; doi:10.1097/MD.0000000000044101)
Supplement: Supplementary file 2 [file medi-104-e44101-s002.pdf]

Supplementary Figure 1: Forest Plot of Mendelian Randomization (MR) estimates for the causal effects of 249 metabolic traits on gout risk.

This figure displays the causal associations (beta coefficients) and 95% confidence intervals derived from the primary inverse-variance-weighted (IVW) MR analysis. Each point represents the effect size (IVW β) of one metabolic trait on gout risk, with horizontal lines indicating confidence intervals. Traits are ordered by effect size. Statistically significant associations (IVW *p*-value < 0.05) are highlighted and correspond to the key findings reported in Table 2 of the main manuscript (e.g., alanine, glycoprotein acetyls, specific lipid ratios). Non-significant associations are shown in gray. The vertical dashed line denotes the null effect ($\beta = 0$). Analyses were adjusted for false discovery rate (FDR) and heterogeneity. Data sources: UK Biobank (exposures) and FinnGen Biobank (outcome).

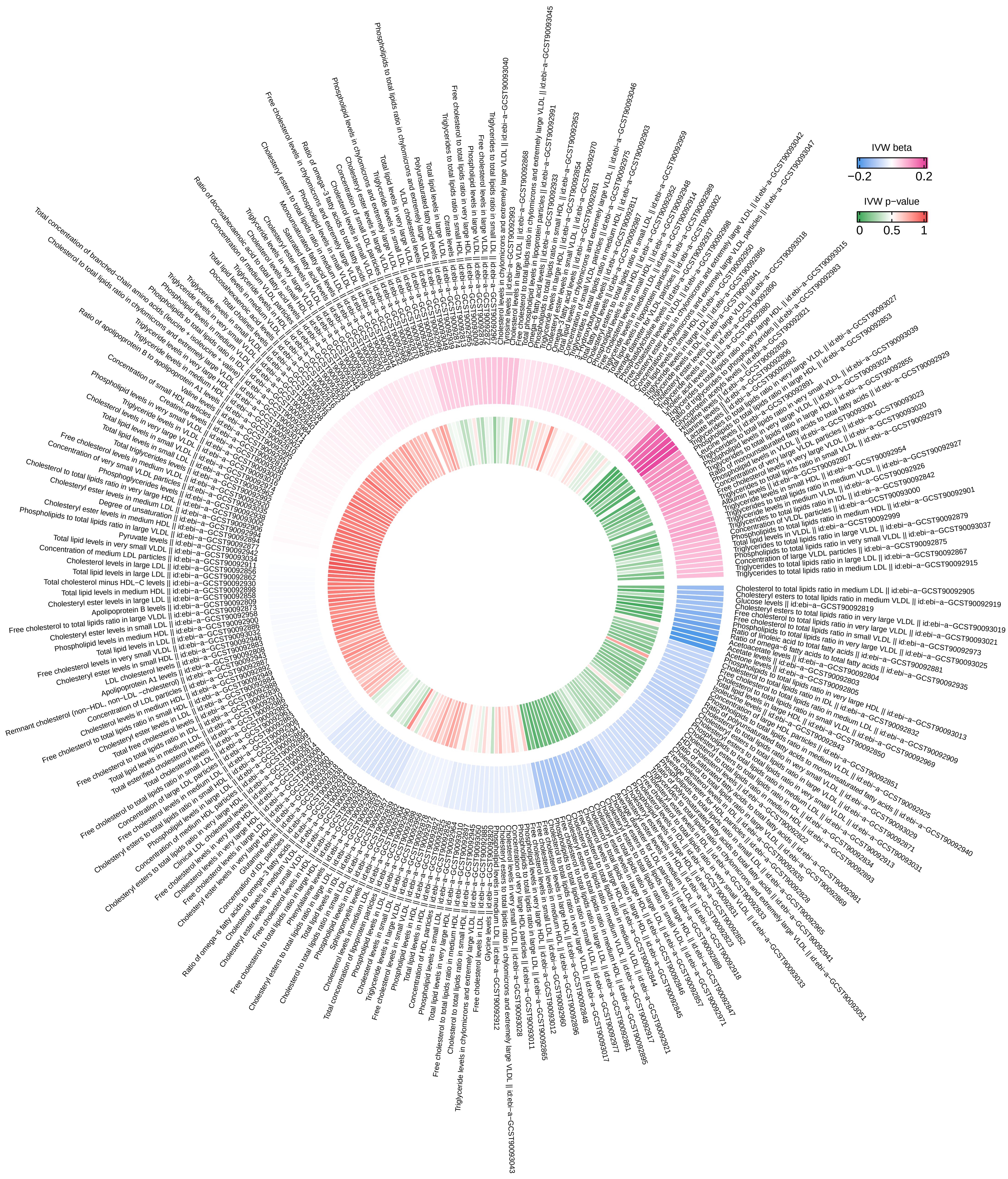
IVW beta
−0.2
0.2
IVW p−value
0
0.5
1
